# Cognitive bias modification for perfectionism and intolerance of uncertainty: A randomized controlled trial

**DOI:** 10.1002/pchj.742

**Published:** 2024-03-07

**Authors:** Kübra Tör‐Çabuk, Volkan Koç

**Affiliations:** ^1^ University of Massachusetts Lowell Lowell Massachusetts USA; ^2^ Istanbul Medeniyet University Istanbul Turkey

**Keywords:** cognitive bias modification, experimental design, intolerance of uncertainty, perfectionism

## Abstract

This study investigated the efficacy of combined cognitive bias modification (CBM) on perfectionism and intolerance of uncertainty. Fifty‐four university students scoring over 70.5 on the Brief Symptom Measure were randomly assigned to experimental and placebo control groups. The CBM intervention was administered online for 4 weeks. Assessments were given at baseline, after the 4‐week intervention, and 1 month post‐intervention. Results showed a statistically significant decrease in two dimensions of perfectionism, concern over mistakes and parental criticism, and intolerance of uncertainty of those in the experimental group, compared to those in the control group. The findings related to the interpretation of perfectionism revealed a significant interaction effect of time and direction of sentences for the experimental group. Lastly, the experimental group's interpretation bias scores for intolerance of uncertainty showed a statistically significant increase after the intervention compared to those in the control group. The study's findings provide preliminary support for the effectiveness of CBM on perfectionism and intolerance of uncertainty.

## INTRODUCTION

Transdiagnostic variables have gained popularity in the mental health field, with an increasing number of interventions having been developed to target these variables (Boswell et al., 2013; Ellard et al., 2010; Fairburn et al., 2003; Khakpoor et al., 2019). Cognitive bias modification (CBM) is among these interventions and aims to systematically change the biases in cognitive processing using experimental paradigms. CBM has previously been used for perfectionism and intolerance of uncertainty, the transdiagnostic variables that play crucial roles in the mechanisms of several psychological disorders (Dodd et al., 2019; Li et al., 2021; Yiend et al., 2011). The current study aimed to use a combined CBM for perfectionism and intolerance of uncertainty.

Perfectionism has been constructed as a multidimensional variable (Frost et al., 1991) and is associated with a wide variety of psychological disorders, including depression, anxiety, eating disorders, and obsessive‐compulsive disorder (Blatt et al., 1995; Chik et al., 2008; Sutandar‐Pinnock et al., 2003). Research suggests that any intervention on perfectionism may affect the efficiency of these disorders' treatment processes (Bieling et al., 2004). Cognitive behavioral approaches are the most widely used treatments for perfectionism (Flett & Hewitt, 2008) and generally utilize the clinical perfectionism model developed by Shafran et al. (2018). The model proposes that perfectionistic individuals tend to interpret real or imagined failures negatively, leading them to experience adverse psychological consequences (Howell, 2017). To date, only a few studies have focused on interpretation bias in perfectionism (Dodd et al., 2019; Howell et al., 2019; Yiend et al., 2011), and the results from these studies indicate that perfectionists interpret ambiguous scenarios more negatively than non‐perfectionists (Howell et al., 2019; Yiend et al., 2011).

Intolerance of uncertainty has been defined as intolerance of ambiguity, novelty, and unexpected changes (Obsessive Compulsive Cognitions Working Group [OCCWG], 1997). Intolerance of uncertainty is closely related to anxiety (Dugas et al., 2004; McEvoy & Mahoney, 2012) and thus is one of the most important risk factors for generalized anxiety disorder (Freeston et al., 1994; Ladouceur et al., 2000). Additionally, it has been suggested that intolerance of uncertainty is closely related to obsessive‐compulsive disorder and is one of the six dimensions underlying the disorder (OCCWG, 1997). Intolerance of uncertainty has also been defined as a cognitive bias affecting the individual's cognitive, emotional, and behavioral reaction to uncertain situations (Dugas et al., 2005). As can be understood from this definition, cognitive biases experienced in information processing are processes that cause intolerance of uncertainty. One study found that people with a high intolerance of uncertainty remember words containing uncertainty more than those with a low intolerance of uncertainty; this reveals that people with a high intolerance of uncertainty find uncertain situations more threatening than those with a low intolerance of uncertainty (Dugas et al., 2005). Therefore, addressing cognitive biases is important in the treatment of disorders related to intolerance of uncertainty.

### Cognitive bias modification (CBM)

Computer‐based studies on systematically modifying cognitive biases are called CBM studies (Koster et al., 2009). In these studies, participants are guided to perceive the given stimulus as desired (i.e., neutral or positive) and not as undesirable (i.e., negative or threatening; Fodor et al., 2020). CBM differs from other interventions in that it is easily accessible, takes less time, and can be delivered using a computer‐based program. Additionally, CBM interferes with relatively automatic cognitive bias processes more quickly and in a more game‐like style, unlike therapy. In cognitive behavioral therapy, the process proceeds in the form of the client finding their automatic thoughts and realizing their cognitive biases, while interventions with cognitive biases occur automatically in CBM (Beard & Peckham, 2020). This paradigm has been increasingly investigated in recent years and studied with several disorders such as depression (Pictet et al., 2016), generalized anxiety disorder (Mathews & MacLeod, 2002; Mineka, 2004), social anxiety disorder (Beard & Amir, 2008), eating disorder (Yiend et al., 2014) and obsessive‐compulsive disorder (Beadel et al., 2014).

In the last 15 years, many studies have been conducted to investigate the effectiveness of CBM on various psychological disorders and transdiagnostic variables (Gonsalves et al., 2019). Recently, many researchers have turned their attention to transdiagnostic variables instead of the diagnostic categories in the *Diagnostic and Statistical Manual of Mental Disorders‐5th Edition* (DSM‐5; American Psychiatric Association [APA], 2013) and the *International Classification of Diseases‐11th Edition* (ICD‐11; World Health Organization [WHO], 2019; Meidlinger & Hope, 2017). Transdiagnostic approaches are preferred both because they are effective in the treatment of conditions where multiple psychological disorders are seen together, as well as because they offer less costly and more practical methods. Perfectionism is one of the transdiagnostic variables emphasized while investigating many disorders' mechanisms (Egan et al., 2011). Likewise, intolerance of uncertainty is one of the transdiagnostic variables that are effective in the emergence and persistence of anxiety‐related disorders (Carleton, 2012; Jacoby, 2020).

Existing studies on CBM for perfectionism and intolerance of uncertainty present promising results (Dodd et al., 2019; Li et al., 2021; Oglesby et al., 2017; Yiend et al., 2011). Yiend et al.'s (2011) study, for instance, examined the relationship between perfectionism and interpretation bias and the effectiveness of CBM for perfectionism. According to their results, the participants who received the cognitive bias modification‐interpretation (CBM‐I) intervention for perfectionism were revealed to make more non‐perfectionist interpretations after the intervention compared to the participants in the control group. Their study was important because it was the first to show perfectionism and interpretation biases to be closely related, and CBM intervention for perfectionism also to be effective on the interpretation biases of people with high perfectionism. The results from Dodd et al.'s (2019) study parallel those of Yiend et al. (2011). The result from Dodd et al.'s (2019) research found that the participants in the experimental group were more inclined to make non‐perfectionist interpretations compared to those in the control group.

CBM has also been used for intolerance of uncertainty (Li et al., 2021; Oglesby et al., 2017). Oglesby et al. (2017) found a significant decrease in interpretation bias for intolerance of uncertainty in the participants who received the CBM‐I intervention compared to those in the CBM‐I control group. Additionally, the intervention decreased the participants' self‐reported scores for intolerance of uncertainty (Oglesby et al., 2017). Their results showed that a single‐session CBM‐I intervention led to significant changes in intolerance of uncertainty and interpretation bias toward intolerance of uncertainty. Li et al. (2021) used a four‐session CBM‐I intervention for intolerance of uncertainty. The intervention program consisted of four sessions and used the word‐sentence association paradigm, as in the study by Oglesby et al. (2017). As a result of Li et al.'s (2021) research, the scores for intolerance of uncertainty from the group that received the CBM‐I intervention decreased significantly compared to those from the control group (Li et al., 2021). At the same time, no change was observed in the anxiety scores of the participants in the control group before and after the intervention. In contrast, the anxiety scores of the participants in the experimental group decreased significantly. In addition to examining the effectiveness of the intolerance of uncertainty‐focused CBM‐I intervention, their study also examined whether intolerance of uncertainty predicts the relationship between this intervention and anxiety. As a result of the multi‐level linear analysis, they found intolerance of uncertainty to partially predict the relationship between anxiety and the CBM‐I intervention.

### Purpose of the study

Although CBM studies have been conducted on transdiagnostic variables such as perfectionism and intolerance of uncertainty, these studies should be repeated in different settings and samples to generalize these results. Additionally, none of these studies used a combined intervention for perfectionism and intolerance of uncertainty. Given the pivotal roles these two transdiagnostic variables play in the mechanisms of several psychological disorders, including depression (Blatt et al., 1995), obsessive‐compulsive disorder (OCCWG, 1997), and eating disorders (Shafran et al., 2018), conducting a combined CBM study can yield valuable insights into their treatment within the context of these disorders. The motivation for focusing on perfectionism and intolerance of uncertainty in this study stems from their significant roles in the development and persistence of various psychological disorders, including obsessive‐compulsive disorder and eating disorders (Pinto et al., 2019; Shafran et al., 2018).

In the current study, the effectiveness of combined CBM‐I on perfectionism and intolerance of uncertainty was investigated with a sample of university students. The main purpose of this study was to examine the effectiveness of a combined CBM‐I for perfectionism and intolerance of uncertainty. Another aim of the study was to test the effectiveness of a CBM study in Turkey. Turkey, as a Middle Eastern country, possesses unique characteristics distinct from Western populations, where most of the CBM research has been conducted. Hence, it is imperative to thoroughly evaluate the effectiveness of these approaches within Eastern populations before considering their widespread adoption. Until now, only one study was found to have used CBM in Turkey. The results of that study on university students with high social anxiety levels were promising regarding the use of CBM in Turkey (Koç & Işıklı, 2021). Different studies to be carried out at this point will allow for the effectiveness of these interventions to be tested and make the intervention more recognizable and applicable to different samples and disorders. Therefore, the current study aimed to extend existing studies on CBM by (1) using a sample of university students in Turkey; (2) using a four‐session CBM‐I intervention; (3) conducting a combined intervention for perfectionism and intolerance of uncertainty; (4) collecting data pre‐intervention, post‐intervention, and 1 month after the intervention as a follow‐up; and (5) including a Brief Symptom Measure (BSM) to test the symptoms of different psychological disorders before and after the intervention given that both variables in the study are risk factors for several psychological disorders.

## METHOD

### Study design

The current study used a 2 × 3‐factorial mixed design with two groups (experimental and placebo control) and three time points (pretest, posttest, and follow‐up). Participants were randomly assigned to two groups: experimental and placebo control. The data collection and four‐session intervention were carried out online. Data were collected at three points in time: before the intervention (T1), after the intervention (T2), and 1 month after the intervention (T3).

### Participants

The study sample consisted of 54 university students. The inclusion criteria are as follows: (1) being over the age of 18; (2) having a score > 70.5 on the BSM at T1 (the cut‐off score is based on the Turkish adaptation of the scale [Gülüm & Soygüt, 2019]); (3) being an undergraduate student; (4) being fluent in Turkish; and (5) being able to access the internet by computer. Participants with any psychiatric disorder or receiving psychiatric/psychological treatment were excluded from the study. Of the 54 participants, 43 (79.6%) were female, and 11 (20.4%) were male. Ages ranged between 18 and 29, with a mean of 21.63 (*SD* = 2.09).

A power analysis conducted using the program G*Power 3.1 (Faul et al., 2009) for a fixed effect one‐way analysis of variance (ANOVA) revealed that a minimum sample size of 28 participants is required to achieve a statistical power of at least 0.8, while maintaining a significance level of .05 and assuming a medium effect size based on previous studies (*d* = 0.5). Anticipating a dropout rate of 30% (0.3), as observed in a previous study conducted in Turkey (Koç & Işıklı, 2021), the sample size, accounting for potential dropouts, was calculated to be 40 participants. A larger participant sample was strategically planned for inclusion to accommodate the exclusion of participants who fell below the cut‐off score or did not complete the study.

### Measures

The Frost Multidimensional Perfectionism Scale (FMPS; Frost & Marten, 1990), the Intolerance of Uncertainty Scale (IU; Freeston et al., 1994), and the BSM (Blais et al., 2015) were administered to the participants at three time points (T1, T2, and T3). Interpretation bias measures for perfectionism and intolerance of uncertainty were used to measure the participants' interpretation biases at the beginning and end of the CBM‐I intervention (T1 and T2).

### Demographics form

Information about the participants' age, gender, and department at the university was obtained during the pretest phase.

### Frost Multi‐dimensional Perfectionism Scale (FMPS)

The Turkish adaptation of the Frost Multidimensional Perfectionism Scale was used to measure the participants' perfectionism symptoms (Frost & Marten, 1990; Kağan, 2011). The scale is a 5‐point Likert‐type self‐report scale with items scored between 1 (*strongly disagree*) and 5 (*strongly agree*). The scale contains 35 items and consists of the six sub‐dimensions of concern over making mistakes, high personal standards, perception of high parental expectations, perception of high parental criticism, doubt over actions, and order and organization. The scale has a high level of reliability in the Turkish sample, with internal consistency for all items being calculated as .91. The internal consistency coefficients of the subscales vary between .64 and .94. The six‐factor structure of the scale was also revealed to be valid as a result of the confirmatory factor analysis (Kağan, 2011). Not including the order and organization sub‐dimension is recommended when calculating the total score. As a result, the total score is obtained by adding the scores for the remaining 29 items and can vary between 29 and 145.

Cronbach's alphas of the sub‐dimensions for this study were .89 for concern over making mistakes, .77 for high personal standards, .87 for the perception of high parental expectations, .83 for the perception of high parental criticism, .79 for doubting the quality of one's actions, and .90 for order and organization.

### Intolerance of Uncertainty Scale (IU)

The Intolerance of Uncertainty Scale was used to measure the participants' symptoms of intolerance of uncertainty. The original scale is in French and was developed by Freeston et al. (1994). The scale was later adapted into English by Buhr and Dugas (2002). The 27‐item scale includes 5‐point Likert‐type items (1 = *Does not describe me at all*, 5 = *Describes me completely*). The Turkish adaptation of the Intolerance of Uncertainty Scale was made by Sari and Dağ (2009). Factor analysis revealed a four‐factor structure: uncertainty is stressful and upsetting; unexpected events are negative and should be avoided; being uncertain about the future is unfair; uncertainty leads to the inability to act (Buhr & Dugas, 2002). The fifth factor, which only included one item, was dropped from the scale since the item‐total correlation was lower (*r* = 0.29). The internal consistency coefficients for the scale's factors were .88, .79, .79, and .79. The total score for the scale is calculated by adding the scores obtained from the 26 items. The consistency of the scale was found to be statistically significant using the test–retest method (*r* = 0.51–0.67, *p* < .01). Cronbach's alpha for this study was .95.

### Brief Symptom Measure (BSM)

The BSM was used to measure participants' general psychological symptom levels (Blais et al., 2015). The scale was developed in English by Blais et al. (2015) as a 25‐item 7‐point Likert‐type scale (0 = *none*, 6 = *extreme*). The internal consistency of the scale was found to be .92. Gülüm and Soygüt (2019) carried out the Turkish adaptation of the scale. The cut‐off score for the Turkish version of the scale was 70.5. A receiver operator curves (ROC) analysis was conducted to calculate the cut‐off point (Gülüm & Soygüt, 2019). This study determined the scale's internal consistency as .92. The scale can also successfully distinguish between clinical and non‐clinical samples. The total score for the scale is determined by adding the scores obtained from the 25 items. Cronbach's alpha for the scale in the current study was found to be .93.

### Recognition ratings test

The recognition ratings test was used to measure the participants' interpretation biases toward perfectionism. The method was first used by Mathews and Mackintosh (2000). The current study uses the recognition ratings test developed by Dodd et al. (2019). The recognition ratings test was given in the first week before the intervention and at the end of the intervention in the last week. During the test, participants were instructed to rate four sentences for ten scenarios. The recognition ratings test starts with a title that is given for each scenario (e.g., SWIM MEET), after which the participant is asked to complete the missing letters in the last word of the sentence (e.g., pl_c_). Once the participant completes the missing letters, a comprehension question is given to confirm whether the participant has understood the sentence. After the participant has read the 10 scenarios and answered the comprehension questions in this way, simple math problems are given that take about 2–3 min to answer. These procedures are given to allow the participant to respond to the next sentences in a way that reflects their interpretation, not just the effect of what is left in their memory. Next, the participant is presented with four sentences that will measure their interpretation of the scenarios in random order. The title of the scenario is shown to the participant along with each sentence. The participant is asked to rate how similar the four sentences given at this stage are to the scenario from 1 (*very different*) to 4 (*very similar*). Each sentence about the scenario has one of (1) non‐perfectionist content (positive target sentence, PT; “As you raise your head above water to see how you did, you are happy with your accomplishment”), (2) perfectionist content (negative target sentence, NT; “As you raise your head above water to see how you did, you feel disappointed that you failed to get first place”), (3) no relation to perfectionism and positive content (positive foil, PF; “As you raise your head above water to see how you did, you hear the audience cheering loudly”), or (4) no relation to perfectionism and negative content (negative foil, NF; “As you raise your head above water to see how you did, your goggles pinch uncomfortably at your face”).

The reliability coefficients of the recognition ratings test in the current study were .75 for PT, .77 for NT, .86 for PF, and .85 for NF in the pretest measurement and .86 for PT, .86 for NT, .89 for PF, and .88 for NF in the posttest measurement.

### Word‐sentence association paradigm

The word‐sentence association paradigm was used to measure the participants' interpretation biases toward intolerance of uncertainty (Beard & Amir, 2008). The materials used during the measurement and intervention were developed by Oglesby et al. (2017). The interpretation bias test was delivered before the intervention in the first week and at the end of the intervention in the last week, just as in the measurement of interpretation bias for perfectionism.

The measurement process consists of four stages. First, a plus sign ‘+’ is displayed on the screen for 500 ms to draw the participant's attention to the screen and indicate the start of the experiment. Then, an ambiguous word or group of words appears on the screen for 1000 ms. A sentence then appears after the group of words. Finally, the participant is asked to indicate whether the word and sentence are related by pressing the right arrow on the keyboard for relevant or the left arrow for irrelevant. In the pretest phase, the participants completed 40 trials. In the posttest phase, another 40 trials were given to the participants that were different from the word‐sentence groups used in the pretest and intervention. A participant gets 1 point for choosing a negative sentence to be irrelevant or a neutral sentence to be relevant after each ambiguous word/group and 0 points for choosing the opposite. The interpretation bias total score for intolerance of uncertainty is determined by averaging the scores obtained from each of these 40 trials, with a total score closer to 1 indicating the participant has less interpretation bias toward intolerance of uncertainty. The reliability coefficient for the cognitive bias measure for intolerance of uncertainty in the current study was .69 for the pretest and .81 for the posttest.

### Procedure

Participants were recruited through social media platforms via an invitation link. The link contained a consent form the participants had to read and approve that summarizes the research's ethical principles; the participants were also required to supply an email address for receiving further information about the study. A total of 178 students accepted the invitation to participate in the research and provided their e‐mail addresses. Of the 178 participants, 110 completed the baseline measures (FMPS, IU, and BSM). Of these, 54 participants with a BSM score > 70.5 were randomly assigned to the experimental group or the placebo control group.

Participants were blind to their assigned group and had ID numbers created by random.org that also allowed the author to be blind to participants' assigned groups.

After the baseline assessment, a link to the practice session was sent to the participants allocated to the intervention. They were asked to complete the session within 2 days to avoid time variations among the participants. All interpretation bias measures and intervention sessions were delivered using the program PsychoPy (version 3.0; Peirce, 2007). Before the intervention's first session, participants completed the baseline measures of interpretation bias for perfectionism and for intolerance of uncertainty. The intervention lasted for 4 weeks. The researcher sent invitation e‐mails for the intervention sessions every week, reminding them to complete them in 2 days and thanking them for their participation.

**FIGURE 1:**
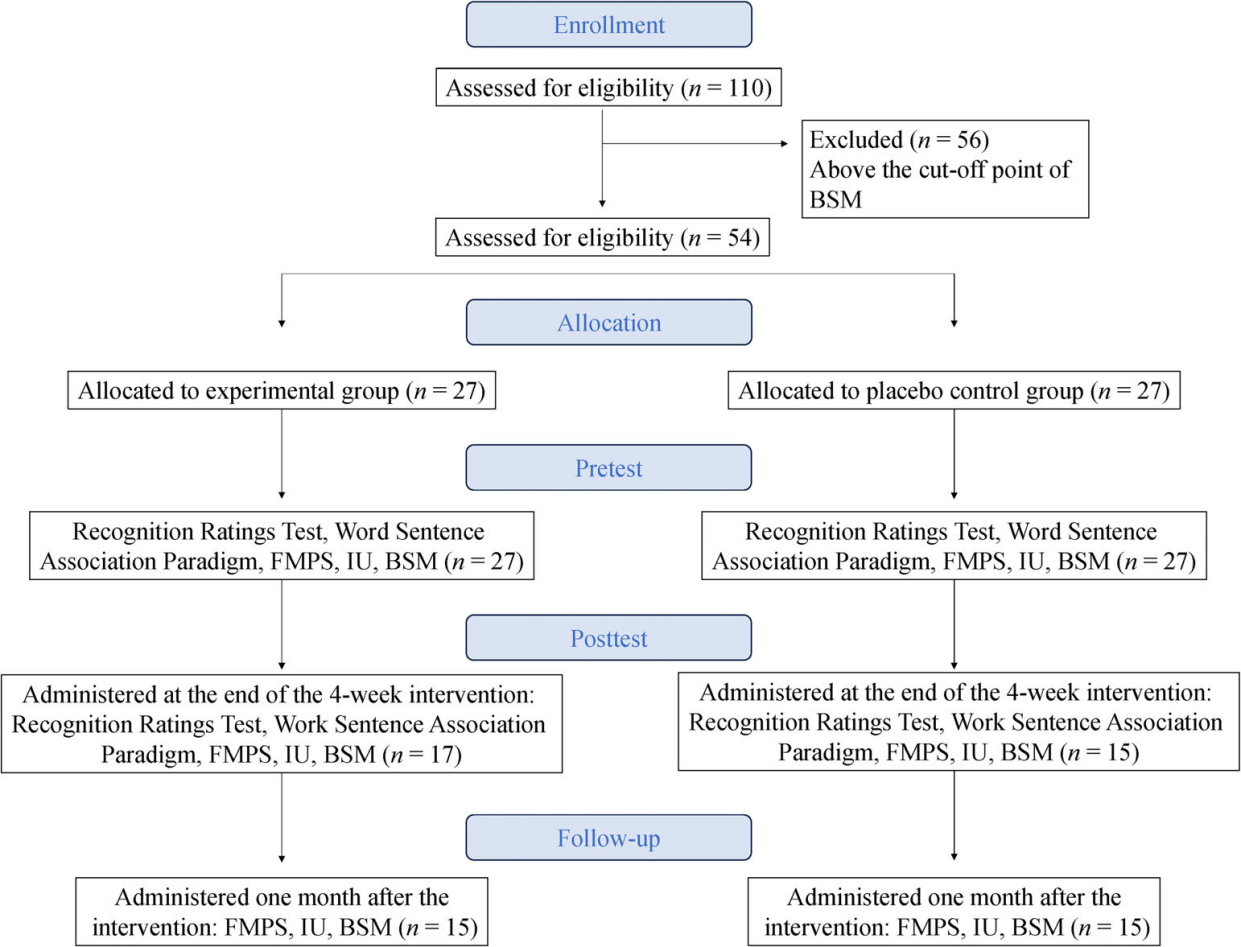
Flow chart of the study. BSM, brief symptom measure; FMPS, Frost Multidimensional Perfectionism Scale; IU, intolerance of uncertainty.

At the end of the fourth session, participants completed the posttest assessments of the interpretation bias for perfectionism and intolerance of uncertainty. Sessions that included baseline and posttest assessments of interpretation bias took approximately 20–25 min. The second and third sessions took an average of 10–15 min. After the final session, the posttest questionnaires were sent to the participants who had completed the 4‐week intervention. A total of 32 people completed the posttest measurement phase. One month later, participants completed the follow‐up questionnaires (FMPS, IU, and BSM). Thirty participants who completed the follow‐up questionnaires were included in the analyses (see Figure 1 for the flow chart of the study). Participants who completed all phases of the research either received additional course credits or participated in a 100 TL gift card lottery to be won by five people.

### Cognitive Bias Modification program

The CBM‐I intervention consisted of four sessions completed at home via the program PsychoPy (v3.0; Peirce, 2007). The links created through the program were sent weekly to the participants. Participants participated in the sessions from their own computers. At the beginning of each session, each participant was expected to write the six‐digit number they had been allocated. Although the intervention proceeded through the same paradigms in the experimental and placebo control groups, the scenario, word‐sentence pairs, and feedback differed for the two groups. The groups underwent the two cognitive bias interventions in a randomized sequence, facilitated by implementing a designated loop function within the PsychoPy program.

### Experimental group

The word‐sentence association paradigm was used in the interpretation bias intervention for intolerance of uncertainty, just as in the measurement of interpretation bias for intolerance of uncertainty. Unlike the measurement process, feedback was given to the participants during the intervention phase. After giving the instructions for the session, a plus sign ‘+’ appeared on the screen for 500 ms to draw the participant's attention to the screen and notify them that the session had started. After this, the word group and sentence appeared (e.g., “party” and “I may not have anyone to talk to”). The participant was then asked to indicate whether the word and sentence were relevant. Because the participants in the experimental group were asked to find negative sentences irrelevant and neutral sentences relevant, feedback was given to the participant in this direction. The participant received “correct” as feedback upon identifying a negative sentence as irrelevant or a neutral sentence as relevant. However, the participant received “incorrect” as feedback upon identifying a negative sentence as relevant or a neutral sentence as irrelevant. The CBM intervention aimed to reinforce the participants' neutral interpretation biases and eliminate their negative interpretation biases. Each week, the participants were given 20 word‐sentence pairs. The word‐sentence pairs used were developed by Oglesby et al. (2017) and translated into Turkish by two different translators.

The ambiguous scenarios paradigm was used in the CBM‐I intervention for perfectionism. Similar to the recognition ratings test, the participant was first given a sentence whose last word was missing some letters. The critical point here was that the word with missing letters would remove the ambiguity from the sentence and make the sentence emotionally meaningful and inclined toward a positive or negative direction upon being filled in. The last word in all the scenarios given to the participants in the experimental group ended in a way that was incompatible with perfectionism. After the participant pressed the necessary keys to complete the missing letters, a comprehension question was asked to reinforce the participant's understanding of the scenario. After the participant answered this question as yes or no, feedback was given to the participants during the intervention portion, which did not happen during the measurement phase. The participant received “true” as feedback upon answering a comprehension question in a non‐perfectionist way or received “false” as feedback upon answering in a perfectionist way. To ensure the participant had comprehended the scenario and to increase the effectiveness of the intervention, the participant was asked to imagine themself in the scenario for 10 s after receiving the feedback. The current study used the scenarios developed by Dodd et al. (2019). The participants were given 10 scenarios in the first and fourth weeks when the pretest and posttest measurements were taken and 20 scenarios in the second and third weeks when no measurements were taken.

### Placebo control group

Similar to the experimental group, the session for the placebo control group started with the interpretation bias intervention for intolerance of uncertainty. Different from the experimental group, the word‐sentence pairs used are given in a way that does not include ambiguous situations. One related example of the word‐sentence pairs used in the placebo control group was Future/Holiday planning, while one unrelated example was Future/A blue car. In the placebo control group and different from the experimental group, half of the scenarios given in the intervention sessions for perfectionism were terminated in a way consistent with perfectionism and half were terminated in a way inconsistent with perfectionism. For example, the scenario given to the experimental group was the same sentence given to the placebo control group: “You and your housemates are ironing your clothes to go out that night. Your housemates miss a few spots on their outfits, but don't seem to mind. Missing some spots while ironing is acceptable to you.” Except for the last words in the scenarios, the placebo control group was given the exact opposite phrases during the feedback phase than was given to the experimental group.

### Data analysis

The data obtained from the pretest, posttest, and follow‐up measurements were used as part of the data analysis, and extreme values were omitted from the analysis. All analyses were conducted using R‐Studio (RStudio Team, 2020). In order to test the effectiveness of the intervention, the 2 × 3‐factorial mixed ANOVA design with the two groups (experimental and placebo control) and three time points (pretest, posttest, and follow‐up measurement) was used for each variable (perfectionism, intolerance of uncertainty, and BSM). The study used the 2 × 2‐factorial mixed ANOVA design with two groups (experimental, placebo control) and two time points (pretest and posttest) for analyzing the interpretation biases toward intolerance of uncertainty, and four‐way mixed ANOVA for interpretation biases toward perfectionism.

## RESULTS

Kurtosis and skewness values reveal whether or not the data are normally distributed in the experimental and placebo control groups and were checked before performing the analyses in order to determine if the parametric tests needed to be applied. The ±2.0 range suggested by George and Mallery (2016) was taken into account for the kurtosis and skewness values and was found to have an acceptable range for all variables.

### Effectiveness of the CBM on perfectionism

**FIGURE 2:**
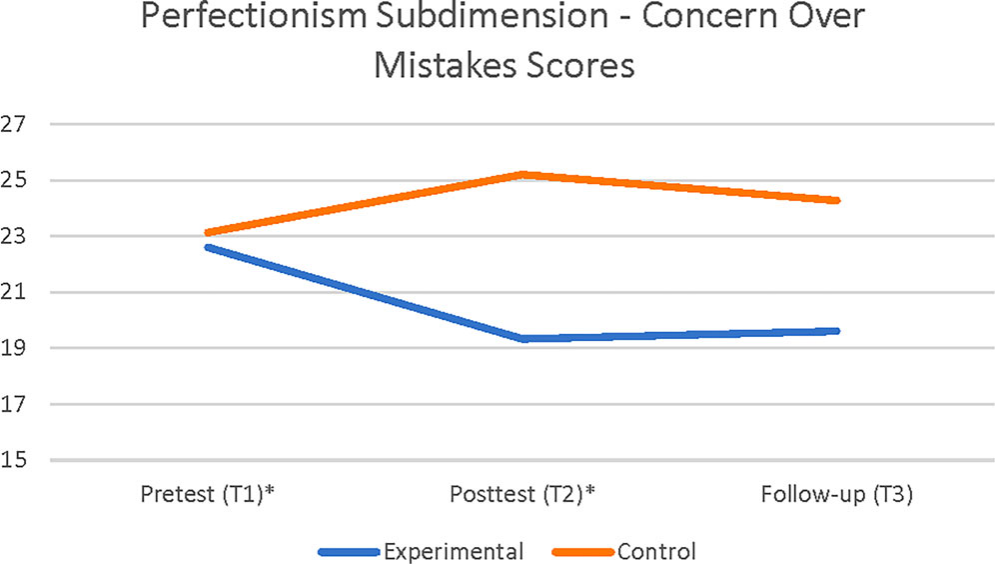
Means for perfectionism outcomes over Time, by study conditions. * indicates significant difference between conditions at *p* < .01.

A 2 × 3‐factorial mixed ANOVA of two groups (experiment and placebo control) and three time points (pretest, posttest, and follow‐up) was conducted to examine the effect of the CBM‐I intervention on the sub‐dimensions of perfectionism. The analysis revealed a statistically significant interaction effect between the CBM intervention and FMPS's first sub‐dimension of concern over making mistakes, Wilk's lambda = 0.75, *F*(2,27) = 4.38, *p* = .032, *η* ^2^ = 0.24. As a result of the contrast analysis, a statistically significant difference was found between the groups regarding the pretest and posttest measurements (*p* = .006), as well as a statistically significant difference between the experimental group's pretest (*M* = 22.60, SD = 8.43) and posttest scores (*M* = 19.33, SD = 5.88). There was no statistically significant interaction effect for FMPS's second sub‐dimension of high personal standards, *F*(2,27) = 0.314, *p* = .580 (see Figure 2). There was no statistically significant interaction effect for FMPS's third sub‐dimension of high parental expectations, *F*(2,27) = 0.845, *p* = .440. A statistically significant interaction effect was found for FMPS's fourth sub‐dimension of perception of high parental criticism, Wilk's lambda = 0.71, *F*(2,27) = 5.45, *p* = .010, *η* ^2^ = 0.28. No statistically significant interaction effect was found for FMPS's fifth sub‐dimension of doubt over one's actions, Wilk's lambda = 0.76, *F*(2,27) = 4.19, *p* = .026, *η* ^2^ = 0.23. Lastly, no statistically significant interaction effect was found for FMPS's last sub‐dimension of order, *F*(2,27) = 0.888, *p* = .201. Table 1 provides the means from the pretest, posttest, and follow‐up measurements for the experimental and control groups' six sub‐dimensions of the FMPS, together with their standard deviations.

**TABLE 1:** Means and standard deviations for the sub‐dimensions of perfectionism.

Sub‐dimensions of perfectionism	Group	Pretest (T1)		Posttest (T2)		Follow‐up (T3)	
		*M*	SD	*M*	SD	*M*	SD
Concern over making mistakes	Experimental	22.60^a^	8.43	19.33^a^	5.88	19.60	8.61
	Control	23.13	7.46	25.20	6.16	24.26	5.93
High personal standards	Experimental	27.13	5.35	27.60	5.34	26.00	5.02
	Control	28.93	5.96	28.46	4.77	28.73	4.97
Perception of high parental expectations	Experimental	15.20	6.30	14.33	6.14	14.73	7.11
	Control	15.53	5.09	15.93	5.68	15.33	5.82
Perception of high parental criticism	Experimental	11.06	5.18	9.60	4.62	10.53	5.62
	Control	10.33	4.38	11.46	4.67	10.80	4.64
Doubt over actions	Experimental	13.33	4.35	12.26	4.18	13.20	4.91
	Control	11.46	3.97	12.86	4.30	12.20	4.31
Order and organization	Experimental	24.60	3.96	22.80	5.40	22.40	5.43
	Control	22.80	5.21	23.80	4.78	22.80	4.70

^a^ Contrast analysis indicated a significant difference between the pretest and posttest of the experimental group, *p* < .01.

### Change in interpretation bias toward perfectionism

A four‐way mixed design ANOVA was conducted for interpretation bias toward perfectionism; factors included Time (pretest, posttest), Sentence Type (target, foil), Direction (positive, negative), and Group (experimental, placebo control). This revealed a significant 3‐way interaction between group, time, and direction; *F*(1,28) = 8.19, *p* = .008, as well as between group, sentence type, direction; *F*(1,28) = 9.75, *p* = .004. There were also significant main effects of Sentence Type, *F*(1,28) = 34.63, *p* < .001 and Direction, *F*(1,28) = 11.25, *p* < .001. Follow‐up two‐way ANOVAs (Direction × Time) were conducted for each group (experimental vs. control). For the control group, the Time × Direction interaction was not significant, *F*(1,28) = .33, *p* = .560, whereas for the experimental group, the same interaction was significant: Time × Direction *F*(1,28) = 5.07, *p* = .030. The main effect of Direction was also significant for the experimental group: *F*(1,28) = 7.26, *p* < .001. Simple main effects were conducted for the Direction (positive vs. negative) of items for the experimental group. There was no significant main effect of the Time for positive items *F*(1,28) = 3.44, *p* = .070. There was also no significant main effect of Time on the negative items for the experimental group *F*(1,28) = 1.73, *p* = .190. The means and standard deviations of the mean similarity ratings are listed in Table 2.

**TABLE 2:** Means and standard deviations from the interpretation bias toward perfectionism measures.

Recognition ratings test	Group	Pretest (T1)		Posttest (T2)	
		*M*	SD	*M*	SD
PT	Experimental	2.66	.56	3.12	.61
	Control	2.59	.54	2.48	.68
NT	Experimental	2.23	.65	1.94	.83
	Control	2.40	.72	2.57	.66
PF	Experimental	1.82	.60	2.23	.89
	Control	2.03	.91	1.91	.80
NF	Experimental	1.81	.74	1.84	.78
	Control	1.90	.88	1.75	.76

*Note*: Recognition ratings ranged from 1 (very different) to 4 (very similar).

Abbreviations: NF, negative foil; NT, negative target; PF, positive foil; PT, positive target.

### Effectiveness of the CBM on intolerance of uncertainty

**FIGURE 3:**
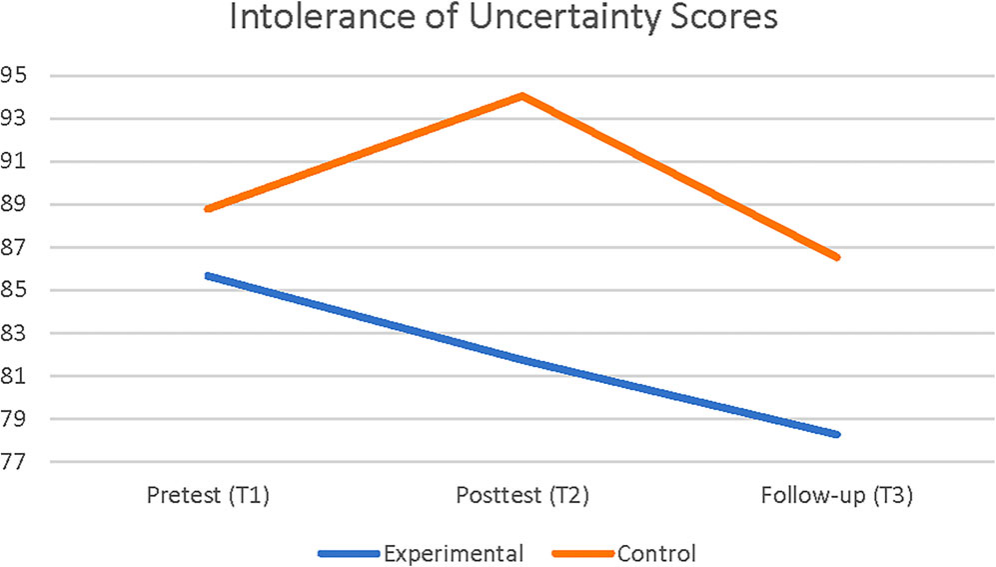
Means for intolerance of uncertainty scores over time, by study condition.

A 2 × 3‐factorial mixed ANOVA with two groups (experimental and placebo control) and three times (pretest, posttest, and follow‐up measurement) was conducted to examine how the CBM‐I intervention changed the scores regarding intolerance of uncertainty. As a result of the analysis, time was found to have a statistically significant effect on the intolerance of uncertainty regarding the CBM‐I intervention, Wilk's lambda = 0.787, *F*(2,27) = 3.64, *p* = .040, *η* ^2^ = 0.21. As shown in Figure 3, the posttest scores from the experimental group (*M* = 81.73, SD = 18.49) were statistically significantly lower than the posttest scores from the control group (*M* = 94.06, SD = 14.31). On the other hand, the CBM‐I intervention was not found to have a statistically significant interaction effect on the measurement of intolerance of uncertainty, *F*(2,27) = 1.50, *p* = .240. Table 3 provides the mean and standard deviations for the groups' pretest, posttest, and follow‐up measurements.

**TABLE 3:** Means and standard deviations for the intolerance of uncertainty measure.

	Group	Pretest (T1)		Posttest (T2)		Follow‐up (T3)	
		*M*	SD	*M*	SD	*M*	SD
Intolerance of uncertainty (IU)	Experimental	85.66	24.03	81.73^a^	18.49	78.26	17.93
	Control	88.80	17.74	94.06^a^	14.31	86.53	13.66

^a^ Experimental condition vs. placebo control condition means differ significantly (*p* ≤ .01).

Abbreviation: IU, intolerance of uncertainty.

### Change in interpretation bias toward intolerance of uncertainty

**FIGURE 4:**
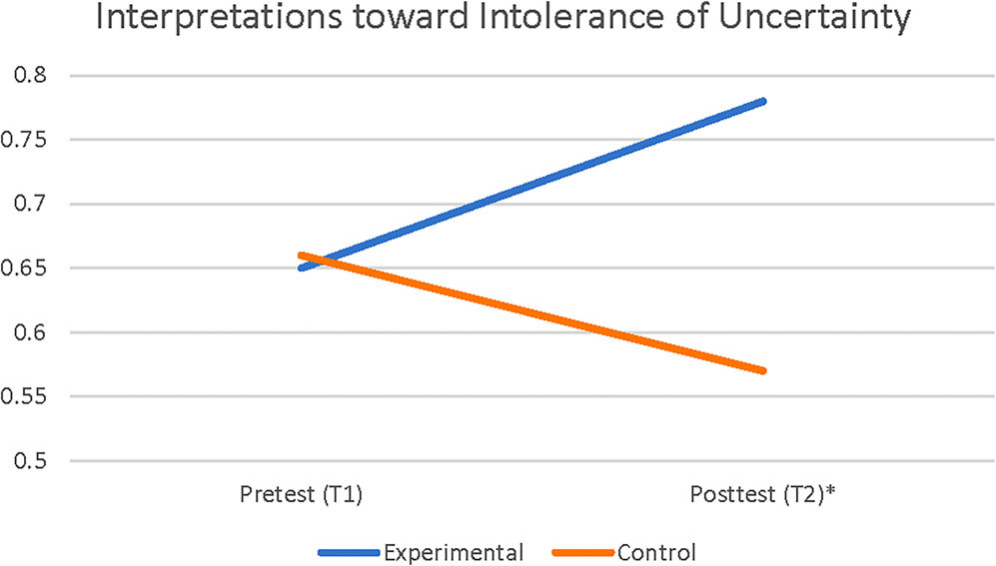
Mean for interpretations bias outcomes for intolerance of uncertainty scores over time, by study condition. * indicates significant difference between conditions at *p* < .01.

A 2 × 2‐factorial mixed ANOVA with two groups (experiment and placebo control) and two time points (pretest and posttest) was conducted to examine how the CBM‐I intervention changed the interpretation bias toward intolerance of uncertainty. The CBM‐I intervention had a statistically significant interaction effect on the intolerance of uncertainty interpretation bias measurement, Wilk's lambda = 0.569, *F*(1,28) = 21.21, *p* < .01, *η* ^2^ = 0.43. When comparing the groups with respect to Time, while no difference is found between the means of the experimental and control groups regarding the pretest measurement, the average score of the experimental group (*M* = 0.78, SD = 0.14) on the posttest measurement was higher than the average score of the control group (*M* = 0.57, SD = .08) (see Figure 4; Table 4).

**TABLE 4:** Means and standard deviations for the interpretation bias toward intolerance of uncertainty measure.

	Group	Pretest (T1)		Posttest (T2)	
		*M*	SD	*M*	SD
IU–Interpretation bias	Experimental	.65	.11	.78^a^	.14
	Control	.66	.10	.57^a^	.08

^a^ Experimental condition vs. placebo control condition means differ significantly (*p* ≤ .01).

### Effectiveness of the CBM on psychological symptoms

A 2 × 3‐factorial mixed ANOVA with two groups (experimental and placebo control) and three time points (pretest, posttest, and follow‐up measurement) was conducted in order to examine how the CBM‐I intervention changed the participants' psychopathological symptom levels according to Time and Group. As a result of the analysis, the CBM‐I was seen to have no statistically significant interaction effect on the scores obtained from the BSM, *F*(2,27) = 0.980, *p* = .759 (Table 5).

**TABLE 5:** Means and standard deviations from the brief symptom measure (BSM).

	Group	Pretest (T1)		Posttest (T2)		Follow‐up (T3)	
		*M*	SD	*M*	SD	*M*	SD
BSM	Experimental	100.80	19.23	101.53	21.20	101.26	28.22
	Control	93.86	21.62	99.40	24.52	96.40	21.56

## DISCUSSION

The current study has investigated the effects of the CBM‐I intervention on two transdiagnostic variables, namely perfectionism and intolerance of uncertainty. FMPS scores revealed a significant interaction effect within the sub‐dimensions of concern over making mistakes and perception of high parental criticism, following the intervention. Specifically, we observed a statistically significant decrease between pretest and posttest scores for these sub‐dimensions, highlighting the effectiveness of the intervention in addressing perfectionism‐related concerns. The CBM‐I intervention also had a significant interaction effect on interpretation bias in perfectionism, together with time and direction effects. There was no significant main effect of the intervention on the direction of sentences. Although the experimental group showed statistically significant changes after the intervention in the mean similarity ratings, these findings should be considered to be only preliminary rather than conclusive.

Regarding intolerance of uncertainty, while the symptom level did not significantly change, there was a significant interaction effect in the interpretation bias measurement. This suggests that the intervention had an impact on how participants interpreted uncertainty‐related scenarios. In addition, the pretest and posttest scores from the experimental and placebo control groups regarding the Word Sentence Association Paradigm (WSAP) were analyzed in order to examine the effect of the CBM‐I intervention on interpretation bias in intolerance of uncertainty. According to the study's hypothesis, the post‐intervention WSAP scores of those in the experimental group were expected to be higher than those in the control group. As a result, the CBM‐I intervention was seen to have a statistically strong and significant interaction effect on the interpretation bias regarding the intolerance of uncertainty measurement. When examining the participants' pretest and posttest data regarding WSAP, the experimental group's posttest scores were found to be statistically significantly higher than the posttest scores from the control group. Additionally, when examining the results from the BSM, no significant interaction effect was found. This could be attributed to the specificity of the intervention scenarios and word pairs used for intolerance of uncertainty and perfectionism.

When looking at the results regarding perfectionism symptoms as one of the variables of the research, the FMPS sub‐dimensions of concern over making mistakes and perception of high parental criticism were found to have significant interaction effects, unlike the other sub‐dimensions. Although there was no significant difference in concern over making mistakes scores between the control and experimental groups during the pretest stage, the experimental group's scores decreased following the intervention. This outcome aligns with prior research, as a study focusing solely on concern over making mistakes found that individuals highly concerned about making mistakes tend to exhibit more interpretation bias related to perfectionism compared to those less concerned (Howell et al., 2019). This underscores the close relationship between concern over making mistakes and interpretation bias in perfectionism, making intervention in this area an expected and effective approach. Furthermore, an analysis of the FMPS's validity and reliability study reveals that concern over making mistakes is the cornerstone of perfectionism (Frost & Marten, 1990). Thus, it is reasonable that the sub‐dimension with the most substantial interaction effect as a result of the intervention was concern over making mistakes.

Another sub‐dimension with a statistically significant interaction effect among perfectionism's sub‐dimensions was the perception of high parental criticism. Two fundamental aspects of perfectionism are a self‐critical attitude and the belief that one's family will be critical in response to failures and mistakes (Shafran et al., 2018). The CBM‐I intervention used in this study incorporated scenarios that reinforced the experimental group's focus on both their accomplishments and their missteps. For instance, participants were presented with a scenario suggesting that achieving a B on an exam would make their parents proud. This interpretation was reinforced by a comprehension question asking whether they would expect their parents to take pride in their grades. Consequently, the scenarios in this study appear to reduce self‐criticism and familial criticism effectively (Dodd et al., 2019).

In order to examine the effect of the CBM‐I intervention on interpretation bias in perfectionism, participants' responses to the recognition ratings test that was used to measure interpretation bias in perfectionism were examined. The hypothesis of the research states that the experimental group's scores on PT, one of the interpretation bias measures in perfectionism, would increase after the CBM‐I intervention. When looking at the studies on perfectionism in which CBM intervention has been used, the most significant change in the measurement of interpretation bias in perfectionism was seen to be in regard to PT (Dodd et al., 2019; Yiend et al., 2011). This measure shows that the participants who received non‐perfectionist intervention interpreted the scenario they had read in a non‐perfectionist way and found a resemblance between the non‐perfectionist positive sentence and the main scenario. Although we could not find a significant main effect of direction (positive vs. negative) or sentence type (target vs. foil) of items after the intervention for the experimental group, there was an increase in the mean similarity ratings of positive sentences from pretest to posttest. On the other hand, although differences were found to have occurred between the groups regarding their scores for NT, PF, and NF, which are the interpretation bias measures in perfectionism, these differences were not statistically significant.

When considering the results related to intolerance of uncertainty, the CBM‐I intervention had no statistically significant interaction effect on the symptom level of intolerance of uncertainty, contrary to the results from existing studies in the literature (Li et al., 2021; Oglesby et al., 2017). Unlike the previous literature, the absence of a change in the symptom level of intolerance of uncertainty in the current study can be explained by factors such as the number of participants, readiness for intervention, and attention, as discussed under the limitations. On the other hand, when examining the results related to the interpretation bias on intolerance of uncertainty, a statistically significant interaction effect was found, in contrast to the symptom levels of intolerance of uncertainty. The current study's results in this direction parallel those of previous studies in the literature (Li et al., 2021; Oglesby et al., 2017).

Finally, when examining the results from the BSM, there was no significant interaction effect. The fact that the study had scenarios and word pairs that were specific to intolerance of uncertainty and perfectionism might have caused a change in those variables but not for other symptoms.

### Limitations and future directions

Hallion and Ruscio (2011) suggested that CBM interventions show effectiveness at the level of bias as opposed to a change at the symptom level. This situation seems likely when the following factors are present: a short‐term intervention, an intervention that occurs online, and participants who are willing to participate. Meanwhile, the fact that the participants did not seek active treatment during the research was an expected result and would have reduced the effect of the intervention they received. Future studies would benefit in this regard from including methods that will increase subjects' participation in the intervention. Moreover, conducting the current study online may have decreased the number of participants involved in the intervention. While the number of participants had been 54 for the pretest measurement, this number decreased to 30 for the follow‐up measurement. Considering that the sample consisted of university students and they already spend a lot of time in online environments due to online courses and exams, having the intervention online could affect the process.

On the other hand, although 28 participants were predicted to be sufficient as a result of the power analysis and based on the effect sizes of previous studies, the current number of participants can be said to have been insufficient for measuring the effectiveness of the intervention and determining its power. A low sample number causes the statistical analyses to fail to have a significant effect, regardless of the difference between the groups' means. For this reason, increasing the number of samples is another point to consider in future studies, as this will increase the statistical power.

When examining the literature related to both perfectionism and intolerance of uncertainty, both variables have been cited as relatively permanent and characteristic personality traits (Budner, 1962; Dugas et al., 1998; Frenkel‐Brunswik, 1949). The fact that both variables are permanent personality traits may lead to the inability to get a quick result from the interventions on these variables. Because the rate of perfectionists who benefit from treatment is known to be affected by their perfectionist personality traits (Blatt et al., 1995), a 4‐week program may not be long enough to result in a significant change in perfectionist symptoms. In this respect, the significant changes seen in some sub‐dimensions of perfectionism in the study show the CBM‐I intervention to have caused some changes in a short time, even on relatively permanent characteristics, and this is promising for future studies.

When looking at the current study's results, the experimental group's posttest scores for the perfectionist sub‐dimensions of concern over making mistakes and perception of high parental criticism showed a statistically significant change compared to the pretest scores. Likewise, the posttest measurement for intolerance of uncertainty showed the experimental group's mean score to be statistically significantly higher than the control group's mean score. However, these results were not consistent in the follow‐up measurement. The existing literature about the effectiveness of CBM interventions shows similar results. Similarly, even though Koç and Işıklı's (2021) CBM study conducted on university students in Turkey showed the scores of the experimental group in the posttest measurement to have a statistically significant difference compared to the control group, this change could not be preserved in the follow‐up measurement.

A decrease in the intolerance of uncertainty interpretation bias was additionally found to have occurred, but no decrease occurred regarding the symptoms of intolerance of uncertainty. The word‐sentence association test used in the intolerance of uncertainty intervention may have had an effect in this regard. As Beard and Peckham (2020) stated, an intervention is expected to affect the symptom level as much as the person imagines themself in more scenarios and spends extended lengths of time in ambiguous test scenarios. However, the short structure of words and sentences in the word‐sentence association test does not allow for this. Future studies can develop CBM‐I interventions that use different paradigms related to intolerance of uncertainty. In addition, while a significant interaction effect was observed in the intolerance of uncertainty interpretation bias measurement, this change did not cause the expected interaction at the symptom level. Also, the word‐sentence pairs used in CBM studies only include word‐sentence pairs for specific areas of life, and this may predictably prevent generalizations to all areas of life. A meta‐analysis study on CBM studies suggested that participants could be asked to write a sentence about the word‐sentence and/or scenario or stop and imagine the scenario and/or word‐sentence pair for a while after the intervention so that the participant can internalize the scenario or word‐sentence pairs used in the intervention and generalize them to their life (Jones & Sharpe, 2017). Future studies may benefit from adding such techniques in which participants can be more active in the process in order to increase the intervention's effectiveness.

Despite the above limitations of the current study, this has been an essential step towards developing and increasing the recognition of an intervention program like CBM that can be delivered quickly, easily, and comfortably. Repeating the study over different samples and with modified paradigms will be useful for retesting the hypotheses examined in this study.

## CONCLUSION

In conclusion, the 4‐week CBM‐I intervention demonstrated effectiveness in addressing bias related to intolerance of uncertainty and perfectionism. While the significance of the intervention's impact varied across different sub‐dimensions of perfectionism and intolerance of uncertainty, this study contributes significantly to the field of CBM research. It is important to note that the findings should be considered exploratory rather than definitive due to the limitations, such as the small sample size and inconsistent effects observed. However, the broader context of CBM research suggests its potential efficacy in addressing various psychological disorders and transdiagnostic variables in recent years (Gonsalves et al., 2019). Given that cognitive bias is a crucial aspect of cognitive therapy, exploring how to modify cognitive bias is essential. In this regard, CBM studies not only provide a swift‐access alternative treatment but also serve as a valuable preparatory phase for cognitive therapy (Yiend & Mackintosh, 2004).

## CONFLICT OF INTEREST STATEMENT

The authors declare no conflicts of interest to be present in the study.

## ETHICS STATEMENT

The ethical approval was provided by the ethical review board of Istanbul Sabahattin Zaim University.
